# The two-cell model of glucose metabolism: a hypothesis of schizophrenia

**DOI:** 10.1038/s41380-020-00980-4

**Published:** 2021-01-05

**Authors:** Dirk Roosterman, Graeme Stuart Cottrell

**Affiliations:** 1 Ruhr Universität Bochum, LWL-Hospital of Psychiatry, Bochum, Germany; 2 School of Pharmacy, University of Reading, Reading, RG6 6AP UK

**Keywords:** Molecular biology, Schizophrenia, Neuroscience

## Abstract

Schizophrenia is a chronic and severe mental disorder that affects over 20 million people worldwide. Common symptoms include distortions in thinking, perception, emotions, language, and self awareness. Different hypotheses have been proposed to explain the development of schizophrenia, however, there are no unifying features between the proposed hypotheses. Schizophrenic patients have perturbed levels of glucose in their cerebrospinal fluid, indicating a disturbance in glucose metabolism. We have explored the possibility that disturbances in glucose metabolism can be a general mechanism for predisposition and manifestation of the disease. We discuss glucose metabolism as a network of signaling pathways. Glucose and glucose metabolites can have diverse actions as signaling molecules, such as regulation of transcription factors, hormone and cytokine secretion and activation of neuronal cells, such as microglia. The presented model challenges well-established concepts in enzyme kinetics and glucose metabolism. We have developed a ‘two-cell’ model of glucose metabolism, which can explain the effects of electroconvulsive therapy and the beneficial and side effects of olanzapine treatment. Arrangement of glycolytic enzymes into metabolic signaling complexes within the ‘two hit’ hypothesis, allows schizophrenia to be formulated in two steps. The ‘first hit’ is the dysregulation of the glucose signaling pathway. This dysregulation of glucose metabolism primes the central nervous system for a pathological response to a ‘second hit’ via the astrocytic glycogenolysis signaling pathway.

## Introduction

The phenotypic complexity and multifarious nature of so-called ‘schizophrenic psychoses’ have prevented a simple, biologically based hypothesis for schizophrenia [1]. The ‘two hit’ hypothesis of schizophrenia suggested back in 2001 by Maynard et al., implied that dysregulation of cell–cell signaling by a ‘first hit’, primes the central nervous system. The ‘second hit’, an environmental insult, promotes a pathological response via the same signaling pathway [2, 3]. Interestingly, environmental insults are common tools used in animal models of fear conditioning or aversive training [4].

Recently, Rowland et al. demonstrated increased l-lactate (l-lac^−^) levels in the brain during acute psychosis [5]. However, they interpreted their novel data using the didactic concept of glucose metabolism that dominates many scientific hypotheses. They assumed that anaerobic glycolysis and mitochondrial dysfunction were the source of the increased l-lac^−^ levels. Here, we integrate the data of Rowland et al. on the basis of the ‘two hit’ hypothesis.

To formulate our hypothesis, we have taken inspiration from Mae Wan-Ho’s voyage of discovery through different areas of contemporary physics, including non-equilibrium thermodynamics, quantum optics, and fractals [6]. We have zoomed in on her sub-cellular map to rearrange the well-established concept of glucose metabolism into a concept of dynamic cell–cell signaling. In the first part of our model, we softly tread the path of glucose in the brain. The second part of our model is much rougher, akin to white water rafting and integrates the dual flow of streams of monocarboxylates.

## An alternative view of glucose metabolism

As scientists, more than a few times in life, we have learnt the pathway of glucose metabolism by rota. But, have the concepts that you have learned changed over the past few decades? When considering this question, bear in mind, that approximately every 9 years the amount of scientific publications double and that glucose metabolism is a scientific field [7]. Also consider that ‘Medizinische Biochemie’ published by Rapoport in 1962 [8] consisted of 992 pages, divided into four sections. Alone, the section dealing with the intermediate metabolism of carbohydrates, lipids, steroids, amino acids and proteins, nucleic acids and porphyrin and haem compounds totaled 336 pages [9]. If we apply the same rationale, the section relating to glucose metabolism must now be worth an extraordinary number of pages, unless nothing has changed in the past 60 years in biology. The belief that nothing has changed and nature has kindly sorted the glycolytic enzymes by the gradual degradation of the carbon backbone can help us to learn the names of the enzymes, products, and substrates. However, it is also one way to avoid the new data presented in this exponential increase in publications. Consider the enzyme, glyceraldehyde 3-phosphate dehydrogenase (GAPDH). A literature search for “GAPDH” and “review” alone generates over 100,000 hits. In biology, glycolysis is known to participate in DNA repair and transcription factor regulation [10], mitochondrial membrane permeability, and apoptosis [11]. Glycolysis is also an on-board engine driving vesicles a distance of up to 1 m [12]. Therefore, simply defining glycolysis as a cytoplasmic process of randomly distributed enzymes providing two molecules of pyruvate and two molecules of NADH-H^+^ is just a man-given definition. Accordingly, it is a matter of definition whether one considers GAPDH as a multifunctional protein with functions distinct from glycolysis, or whether all functions performed by GAPDH relate to glycolysis [13].

One of the consequences of this flood of information is that scientists tend to specialize in small and distinct scientific fields. Fatally, this leads to the repetition of the didactically based concept of glucose metabolism in all scientific manuscripts. As a consequence, the didactic sorting of glycolytic enzymes has changed into a scientific ‘fact’. We are not only questioning the ‘fact’ but also provide an alternative model; glucose-metabolizing enzymes sorted by energy transfer.

Depending on the textbook, GAPDH forms NADH-H^+^, which diffuses as NADH to the mitochondria to be recovered. Alternatively, more complicated cytosolic metabolic steps can be introduced. For example, in the textbook of Rapoport from 1987, GAPDH is functionally linked with lactate dehydrogenase (LDH) [14]. Yet in scientific manuscripts, it depends on the topic whether the GAPDH•LDH complex [15, 16], LDH, and l-lac^−^ exist [17]. In modern didactic biology, LDH does not exist because LDH is only introduced when obligate aerobic organisms start to ferment glucose to lactic acid (l-lacH). A similar process is well known for facultative aerobic yeast fermenting glucose to ethanol. Logically, as the scientific field of glucose metabolism grows exponentially, the didactically based concept of glucose metabolism must decline.

Learning is like rowing against the current. As soon as you stop, you drift backwards. The well-established concept of glucose metabolism starts by learning names of a dozen enzymes arranged by the graded degradation of the carbon backbone. It ends by drawing a circle around these enzymes and naming it the blueprint of life. However, this blueprint of life is a flowchart in a circle and akin to the fairy tale, ‘The Emperor’s New Clothes’. Scientists have been rowing the current concept of glucose metabolism up-stream for centuries. Here we test the hypothesis that learning is like floating the path scientists have rowed. Our floating tour takes us down the ‘glucose creek’ in the brain. Here, when neurons fire and consume fuel, the reverberatory current is the flow of glucose and glucose metabolites. These reverberatory currents induce lasting cellular changes that add to the stability of the current [18]. Put simply, synapses with high-energy turnover stabilize themselves. Growing evidence suggests that the disease group ‘schizophrenic psychosis’ is linked to turbulences in this ‘glucose creek’ [5].

Let us assume the fuel of neurons is a mixture of l-lacH, pyruvic acid (pyrH), and glucose. This turns glucose metabolism into a signaling pathway, because l-lac^−^ and pyruvate (pyr^−^) directly interact with the cellular NAD^+^/NADH-H^+^ ratio. Glucose metabolism is highly dynamic and monocarboxylates, such as pyr^−^ and l-lac^−^ are permanently and simultaneously imported and exported by the same cell. This dynamic sending (export) and receiving (import) of monocarboxylates modulates pH- and redox-sensitive signaling pathways [19]. An imbalance in the NAD^+^/NADH ratio is associated with schizophrenia [20] and the activity of the risk gene, catechol-*O*-methyltransferase depends on the redox status of the cystine group [21]. It is well established that glucose itself directly triggers the release of insulin from pancreatic β-cells [22]. Consequently, glucose metabolism is a signaling pathway and glucose and glucose metabolites are signaling molecules. Furthermore, glycogen granules represent storage vesicles of the signaling molecules, glucose-1-phosphate and glucose. Importantly, memory formation is functionally linked to astrocytic glycogenolysis [4]. In astrocytes, muscle cells and thrombocytes the catabolic breakdown of glycogen is the preferred route of metabolism. The central position of astrocytic glycogenolysis in memory formation suggests that the signal encoding memory formation is encoded in the change of the l-lac^−^/pyr^−^ ratio.

## Following the ‘sweet’ signal

**Fig. 1:**
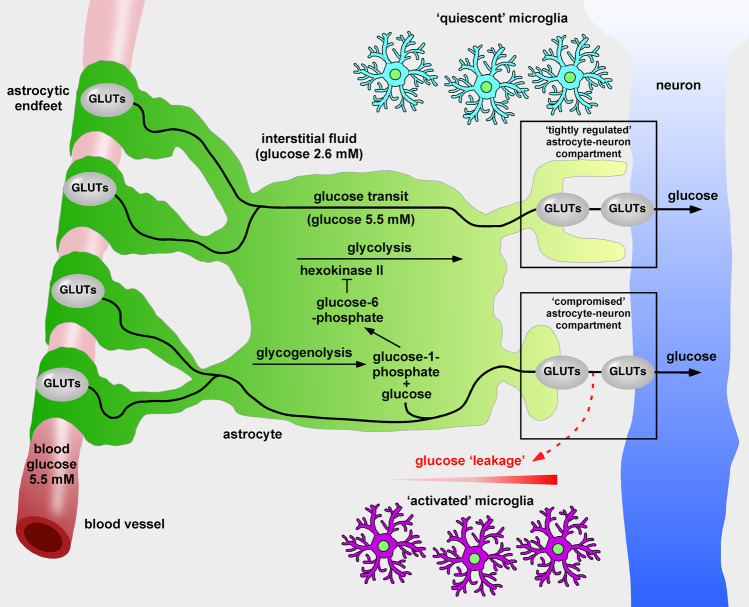
Hypothetical flow of glucose in the brain. The concentration of glucose in the interstitial fluid is low (2.6 mM) [23] and insufficient to supply neuronal compartments of high-energy demand. Astrocytic endfeet completely cover capillaries, where the concentration of glucose is high (5.5 mM) [25]. Glucose is transported via glucose transporters (GLUTs) from the blood stream, across the astrocyte and delivered to neuronal compartments of high-energy demand via astrocytic and neuronal GLUTs. Astrocytic glycogenolysis is a signaling pathway producing glucose, which enters the glucose transit pathway and glucose-1-phosphate. The latter is converted to glucose-6-phosphate, which inhibits hexokinase II [73], thereby blocking astrocytic glycolysis. Decreased astrocytic glycolysis further increases the availability of glucose for neuronal transit. A ‘tightly’ regulated astrocyte-neuron compartment ensures delivery of glucose to the neuron and keep microglial cells in a ‘quiescent’ state. In contrast, a ‘compromised’ astrocyte-neuron compartment, perhaps due to the altered expression of stabilizing genes, genetic variations, or epigenetic modifications, may lead to glucose ‘leakage’, promoting formation of a glucose gradient and subsequent microglial activation.

Our suggested route down the ‘glucose creek’ of the brain is not present in commonly used brain architecture maps (Fig. 1). Consider the glucose concentrations in the brain. In 1980, Gjedden et al. revealed that the concentration of glucose in brain interstitial fluid (ISF) is low (2.6 mM) [23]. More recent data showing that ISF and cerebrospinal fluid are permanently exchanged, support this [24]. The low glucose concentration of the ISF strongly suggests that neurons do not acquire their glucose from the ISF. Instead, astrocytes must provide neurons with glucose and that microglial cells are kept ‘arrested’ in low glucose. Microscopic data demonstrate that blood capillaries (5.5 mM, glucose) are covered by astrocytes, meaning neurons do not have direct access to blood vessels [25]. Furthermore, neurons themselves are not completely covered by astrocytes. Thus astrocytes, through direct and specific contacts with neurons, act as pipeline of glucose to neuronal compartments of high-energy demand [26].

The first glucose transporter (GLUT) was cloned in 1985 [27]. This discovery places GLUTs, rather than hexokinase II as the first regulatory enzyme of glycolysis. As such, our tour down the ‘glucose creek’ means we encounter the first enzyme of glucose metabolism three times. In the context of schizophrenia, the critical passage is the astrocyte-neuron compartment and it is stabilization of astrocyte-neuron compartments that is key. It is obvious, that cell-adhesion proteins must also play a key role in glucose flow [28]. Mechanisms stabilizing the cell–cell compartment, such as mechanisms that accelerate glucose flow are directly involved in regulating the stability of the astrocyte-neuron compartment [29]. Mechanisms that detect an incompletely sealed compartment and mechanisms involved in pruning, brain plasticity, and brain development are also involved in regulating the stability of astrocyte-neuron compartments [29]. Interestingly, many of the listed risk genes and biomarker candidates of schizophrenia are functionally and directly connected to the dynamics of this cell-to-cell passage of glucose [1].

Memory consolidation is functionally coupled to the upregulation of mRNA for proton-linked monocarboxylate transporter 1 (MCT1), muscle LDH (LDH-m), and heart LDH (LDH-h), astrocytic and neuronal GLUTs and astrocytic proton-linked MCT4 [30]. Only the neuronal proton-linked MCT2 remains unchanged [30]. Thus, as a key regulator of glucose flow, the upregulation of neuronal and astrocytic GLUTs causes the ‘glucose creek’ to switch from a trickle to a torrent.

If we understand schizophrenia as an imbalance in glucose metabolism and glucose metabolism is the signaling pathway that dynamically regulates the stability of the astrocyte-neuron compartment. Then it is rational to suggest that an imbalance in glucose metabolism can be linked to dysregulated astrocyte-neuron compartments. A dysregulated astrocyte-neuron compartment can leak the signaling molecule glucose, into the ISF and activate surrounding microglial cells. Glucose-activated microglial cells upregulate inflammatory proteins and increase phagocytosis and tumor necrosis factor-alpha secretion [31]. Interestingly, glucose deprivation sensitizes microglia, promoting the release of inflammatory mediators, and primes microglial function [31]. As discussed by Stöber and co-workers, the release of other biomarker candidates, such as interleukin-1β (IL-1β), IL-6 and brain-derived neurotrophic factor are also directly triggered by glucose or its metabolites [1, 32]. The direct action of glucose and glucose metabolites in regulating cell homeostasis sets glucose metabolism as the ‘major in command’ or as the ‘operating system’ of cells. Thus, the ‘chicken-egg dilemma’ in schizophrenia can be reduced to imbalance in glucose metabolism [33].

The tour down ‘glucose creek’ is too short to take in other details. Data, such as the histology showing reduced numbers of neutrophils, the steroid-dependent regulation of cell-adhesion proteins in context of pruning and schizophrenia, the glutamate-glutamine cycle and the naming and functional integration of further ‘risk’ genes, would surely further support our hypothesis [1]. It would also prevent us from reaching the end of our tour, which concludes with the fact that glucose levels are significantly increased in the cerebrospinal fluid of ‘drug naïve’ schizophrenics [34].

## The two-way flow of monocarboxylic acids

The opening of a bottle of champagne releases a force powerful enough to propel the cork to the ceiling and beyond, a bit like releasing a mysterious Djinn. If a multicellular organism channels such a force (or flow of energy) via a theoretical monocarboxylic acid•carbonic acid exchanger, then oxidative phosphorylation would be coupled to the uptake of its substrate, l-lacH from the environment [35]. In fact, this theoretical monocarboxylic acid•carbonic acid exchanger exists in the form of the proton-linked MCT1•carbonic anhydrase II (CAII) complex [36].

**Fig. 2:**
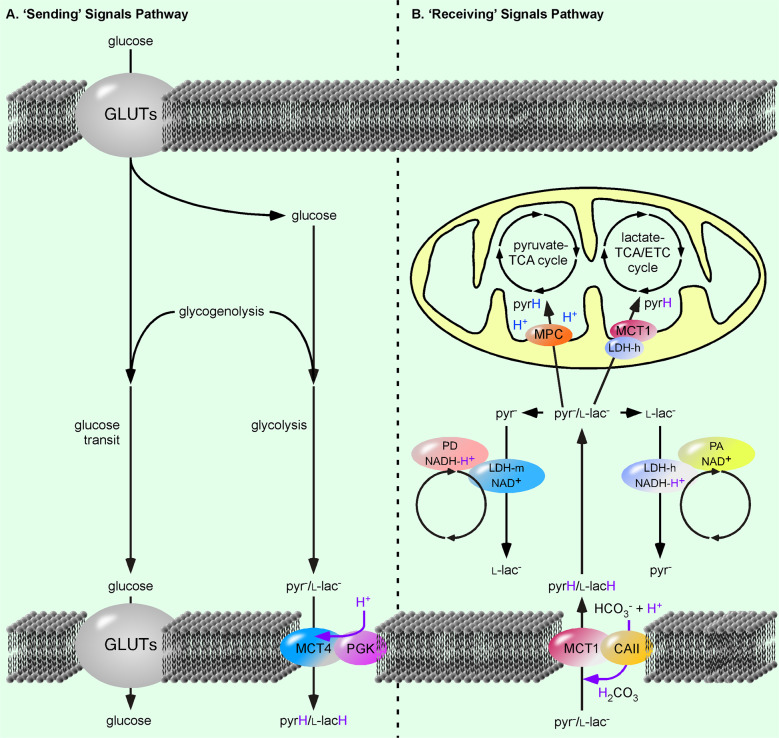
The ‘sending’ and ‘receiving’ of signals via glucose metabolism. **a** Glucose, taken up via glucose transporters (GLUTs) and produced by the breakdown of glycogen (glycogenolysis) is exported as a signal via GLUTs. In addition, glucose is converted to pyruvate (pyr^−^) and lactate (l-lac^−^) via glycolysis and exported as pyruvic acid (pyrH) and lactic acid (l-lacH) via the proton-linked monocarboxylate transporter 4 (MCT4)•phosphoglycerate kinase (PGK) complex [35]. **b** Pyr^−^ and l-lac- are imported as pyrH and l-lacH into the cell via the MCT1•carbonic anhydrase II (CAII) complex [35]. A cytosolic muscle lactate dehydrogenase (LDH-m)•proton donor (PD) complex converts pyruvate (pyr^−^) to lactate (l-lac−) and a cytosolic heart LDH (LDH-m)•proton acceptor (PA) complex converts lactate (l-lac^−^) to pyruvate (pyr^−^). Alternatively, l-lac^−^ is converted to pyr^−^ by the proton-linked mitochondrial MCT1•LDH-h complex to fuel the l-lac^−^-tricarboxylic acid (TCA)/electron transport chain (ETC) cycle [35]. Pyr^−^ is transported into the mitochondrial matrix via the mitochondrial pyr^−^ carrier (MPC) to power the pyr^−^-TCA cycle [35] tit. A violet H^+^ indicates the H^+^ is part of a proton-coupled reaction. The blue H^+^ indicated on the pyrH transferred by the MPC, comes from the mitochondrial matrix.

The well-established flowchart of glycolytic enzymes is in fact a riddle; one molecule of glucose (C_6_H_12_O_6_) is metabolized to two molecules l-lac^−^ (C_3_H_5_O_3_^−^). This is in contrast to the work of Meyerhof, which determined that one molecule of glucose is metabolized to two molecules of l-lacH (C_3_H_6_O_3_) [37]. So, the riddle is, ‘Where have the two protons (H^+^) gone?’. The answer to this conundrum must be that one of the well-established glycolytic reactions is incorrect and that one of the intermediate products of the glycolytic pathway must be an organic acid. If this continuously formed acid is located in the immediate proximity of a proton-linked MCT, a pH gradient (or flow of energy) will be created. This flow of energy will be sufficient to unidirectionally drive the export of monocarboxylic acids (Fig. 2). Importantly this export would be independent from the pH and monocarboxylate concentration of the cytosol and environment. It would, however, depend on glycolytic flow. A proton-linked MCT4•phosphoglycerate kinase (PGK) complex would be perfectly placed to fulfill this task [35]. Unexported monocarboxylic acids would not only increase cytosolic monocarboxylate, they would also generate an additional signal in the form of a proton. This proton would be detected by pH-dependent metabolic adenylyl cyclase and directly couple the glycolysis rate to cAMP-dependent signaling pathways [38]. We would then have a situation where the proton-linked MCT1•CAII unidirectionally imports monocarboxylic acids at the same time in the same cell, as the proton-linked MCT4•PGK complex unidirectionally exports monocarboxylic acids [39]. In this situation, the well-established concept of enzyme kinetics, based on reversibly acting enzymes collapses.

The didactic concept of glucose metabolism inveigles us to consider proton-linked MCTs as l-lac^−^ transporters. However, proton-linked MCTs actually catalyze the membrane exchange of monocarboxylic acids (pyrH and l-lacH). Moreover, by changing the concept of enzyme kinetics, proton-linked MCTs no longer drive equilibrium reactions. Instead they act unidirectionally and pyr^−^ and l-lac^−^ become signaling molecules for distinct mitochondrial and cytosolic enzyme complexes, able to trigger distinct metabolic signaling pathways. The mitochondrial LDH-h•MCT1 complex [40] and the postulated lac^−^-tricarboxylic acid (TCA)/electron transport chain (ETC) cycle [35] led us to consider l-lac^−^ and pyr^−^ as catabolic and anabolic signaling molecules, respectively.

Mitochondrial LDH activity was first suggested by Dianzani in 1951 [41] and later, LDH-h was localized to mitochondria [42]. Over 30 years later, the mitochondrial proton-linked MCT1•LDH-h complex was functionally characterized [40]. The mitochondrial proton-linked MCT1•LDH-h complex oxidizes l-lac^−^ to pyr^−^ and transfers pyrH through the inner mitochondrial membrane. From 1961–1990, L. Reed consistently published that pyrH, not pyr^−^, is substrate of the alpha-ketoacid dehydrogenase, pyruvate dehydrogenase (PDH) complex [43, 44]. It is ironic, that although didactic teaching has set pyr^−^ as the end product of so-called ‘aerobic’ glycolysis, the mitochondrial pyr^−^ transporter, required for the transfer of pyrH into the mitochondria, was not discovered until fairly recently [45, 46]. Thus, whereas in science the l-lac^−^-TCA/ETC cycle was already characterized, the didactic glycolytic flowchart teaches that the product of cytosolic pyruvate kinase is pyr^−^ and that pyr^−^ is the substrate of PDH complex [47]. Thus, for generations, we have learned alchemy, a concept based on faith and not the law of mass conservation.

Our tentative sorting of glycolytic enzymes is helpful for the astrocyte-neuron lactate shuttle hypothesis [48]. However, the concept that l-lac^−^ is food for ‘thoughts’ (memory consolidation) cannot rationally be followed on basis of well-established enzyme kinetics, because here, the uptake of l-lacH is the uptake of a substrate of an anabolic pathway (gluconeogenesis) [49]. Enzyme kinetics, based on concentration-equilibration, predicts that increasing l-lac^−^ is building block for storage and not for consumption. However, combining the import and export of monocarboxylic acids (in sterically separated and distinctly regulated domains) with the mitochondrial proton-linked MCT1•LDH-h complex, means that imported l-lac^−^ must be consumed. l-lac^−^ becomes a substrate for the mitochondrial catabolic pathway. If we consider that the provision of an energy entity (H^+^) predicts the activity of the complex, the activity of LDH-h complexes depends on the l-lac^−^ concentration. Thus, l-lac^−^, generated from either increased glycogenolysis or increased uptake of l-lacH, accelerates ETC activity. Given that the inner-complex stability of NADH-H^+^ is time-dependent, a relative absence of the substrate of the coupled enzymatic reaction is signaled by the release of the ‘second messengers’, NADH and H^+^. In contrast, LDH-m acts as a proton acceptor in enzyme complexes. Here pyr^−^ does not provide the energy entity, H^+^, instead LDH-m accepts NADH-H^+^. Thus, the relative absence of pyr^−^ also predicts the release of the ‘second messengers’.

Whereas increased uptake of l-lacH enhances the ‘catabolic’ signaling pathways via cytosolic and mitochondrial LDH-h complexes, increased uptake of pyrH is detected by ‘anabolic’ signaling pathways, via cytosolic LDH-m complexes and the mitochondrial pyr^−^ transporter. The distinction between pyrH and lacH is made clear in the animal model of exercise-induced hyperinsulinism. Here, the proton-linked MCT1 uptake of pyrH, but not l-lacH, triggers insulin release by pancreatic β-cells [22].

## Schizophrenia treatments: electroconvulsive therapy and olanzapine

The brief tour we have taken down the ‘glucose creek’, together with the exchange of monocarboxylates is sufficient to de-mystify the mysterious mechanism of electroconvulsive therapy (ECT) for treating schizophrenia [50]. Fear conditioning experiments performed using animal models of learning [4] and electroshock therapy, first described in 1756 by Wesley [51], have one thing in common. They both trigger glycogenolysis. Now, by simply changing the point of view and considering the biological function of glycogen granules as a storage of metabolic signaling molecules and cross-referencing with the fact that the scientific field of learning and behavior acknowledges astrocytic glycogenolysis as a key mechanism. We can de-mystify ECT as a mechanism for the nonspecific activation of a signaling pathway; astrocytic glycogenolysis.

Let us consider ECT and sodium-serotonin reuptake inhibitors (SSRIs). ECT causes upregulation of cell surface Na^+^/serotonin symporters in thrombocytes [50]. Thus SSRIs, by blocking the activity of Na^+^/serotonin symporter, act in the diametrical opposite way. There is evidence that Na^+^/serotonin symporter activity is linked to the glycogen storage pool, because SSRIs cause the depletion of glycogen storage in thrombocytes [52]. Furthermore, SSRIs impair thrombocyte hemostatic responses, with bleeding a side effect of SSRI medication. Therefore, it is logical to assume that the depletion of the glycogen pool indicates an imbalance in the metabolic balancing between anabolic and catabolic pathways, in favor of catabolic pathways. There is evidence that SSRIs stimulate astrocytic glycogenolysis [53, 54]. The increase in astrocytic glycogenolysis is a signal that can be conveyed to neurons via supply of glucose and a change in the transfer of monocarboxylates i.e., the l-lac^−^/pyr^−^ ratio. As neurons do not have glycogen stores, they cannot respond to SSRIs by increasing glycogen breakdown. Thus, pharmacological blockage of Na^+^/serotonin symporter activity encodes a signal changing the metabolic balance in astrocytes and neurons in the direction of catabolic pathways. However, although the astrocytic signal impacts the stability and glucose consumption of astrocyte-neuron compartments, SSRIs are only likely to increase presynaptic glucose consumption. This suggests a mechanism for the actions of SSRIs in the treatment of schizophrenia patients [55].

In an organism, the dynamic exchange of the signaling molecules, glucose, pyrH, and l-lacH, allows certain cells, such as muscle and brain cells to adapt or to learn from increased metabolite provision. Pancreatic β-cells participate in detection and regulation, and other cells such as adipocytes, act as storage. It is well known that antipsychotics, such as olanzapine, have a huge impact on glucose homeostasis in humans. Lynch and co-workers determined the transcriptional changes induced by olanzapine treatment on genes related to the metabolism of glucose in rats [56]. If we extrapolated the observed mRNA changes in rat muscle to human astrocytes, pancreatic β-cells, and adipocytes, then we could infer that the decrease of mitochondrial *Slc16a1* and *Ldhb* (genes of the MCT1•LDH-h complex) (Table 1) would decrease mitochondrial l-lac^−^ consumption. The decreased l-lac^−^ consumption would then change the cytosolic l-lac^−^/pyr^−^ ratio, in favor of l-lac^−^. Astrocytic glycogenolysis greatly increases the l-lac^-^/pyr^-^ ratio [17]. This signal encodes the postulated stabilization of the astrocyte-neuron compartment and increases activity of LDH-h complexes. Hereby, astrocytes and neurons learn to consume more glucose by stabilizing the astrocyte-neuron compartment and upregulation of GLUTs [30].

**Table 1:** List of gene and common protein names and abbreviations.

Gene name	Common protein name	Abbreviation in text
*Ca2*	Carbonic anhydrase II	CAII
*Gapdh*	Glyceraldehyde 3-phosphate dehydrogenase	GAPDH
*Hk2*	Hexokinase II	None
*Ldha*	Muscle lactate dehydrogenase	LDH-m
*Ldhb*	Heart lactate dehydrogenase	LDH-h
*Pgk1*	Phosphoglycerate kinase	PGK
*Slc16a1*	Monocarboxylate transporter 1	MCT1
*Slc16a3*	Monocarboxylate transporter 4	MCT4
*Slc16a7*	Monocarboxylate transporter 2	MCT2
*Slc9a1*	Na^+^/H^+^ exchanger 1	None
*Slc9a2*	Na^+^/H^+^ exchanger 2	None
*Slc9a3*	Na^+^/H^+^ exchanger 3	None

We interpret the data of Tadi and co-workers that both astrocytes and neurons adapt their cell homeostasis to a higher glucose turnover. This interpretation contrasts with the astrocyte-neuron lactate shuttle hypothesis, which focuses on l-lac^−^ as food for hungry neurons. Thus, an increase in monocarboxylic acid transfer, here interpreted as astrocyte-neuron communication [39], should entail the upregulation of neuronal proton-linked MCT2, but not the upregulation of neuronal GLUTs.

Increases in *Gapdh* and *Ldha* (GAPDH•LDH-m complex) and *Slc16a3* and *Pgk1* (MCT4•PGK complex) would increase the glycolytic flux in astrocytes and muscles [56, 57]. The decrease in *Hk2* (hexokinase II) in pancreatic β-cells would result in an impaired detection of glucose, giving rise to hyperglycemia [58]. The decrease of *Slc16a1* and *Ldhb* would mean less substrate for the catabolic l-lac^−^-TCA/ETC cycle. This would shift glucose metabolism towards the preferentially anabolic pyr^−^-TCA cycle, which likely depends on the acidic pH of the mitochondrial matrix [59] or alternatively, is driven by mitochondrial pyrH/carbonic acid antiporter. Together, this shift may be responsible for the weight gain in olanzapine-treated patients [60]. Cells not designed for energy storage, would ‘starve’ and switch to free fatty acids as an energy source, even during hyperglycemia [58]. The impact of olanzapine on metabolism was brilliantly shown by the fact that the switch to fatty acid metabolism is required for survival [61].

By drawing on a wealth of information taken from bench to bedside. The presented concept of glucose metabolism fits well as a unifying hypothesis of schizophrenia and explains the observed effects of ECT and pharmacological medication.

## Discussion

The review provides a simple, biologically based hypothesis of schizophrenia. It is sufficient to integrate most aspects of schizophrenia including treatment with ECT or olanzapine. We have deliberately focused on the ‘second hit’ of the astrocytic glycolysis and visited dysregulated glucose metabolism. We have not looked forward and examined the signaling pathways activated by biomarker candidates of schizophrenia, such as tumor necrosis factor-alpha, IL-1β, and IL-6. However, we have looked back and found that glucose and glucose metabolites are a common denominator in the release of these biomarkers.

The presented concept of glucose metabolism is highly original. All the key points regarding glucose metabolism as a signaling pathway already published, but interestingly have not been brought together into one unifying hypothesis. Of course, not all the points highlighted are found in the common understanding of glucose metabolism and are therefore sometimes ignored or rejected by the scientific community. Just suggesting that pyrH, instead of pyr^−^ is the substrate of the mitochondrial PDH complex [43] and illustrating membrane-located PGK at the membrane [62], make this hypothesis unique.

In our discussion of glucose metabolism, we place the mitochondrial proton-linked MCT1•LDH-h as a pH-independent complex, feeding the catabolic l-lac^−^-TCA/ETC cycle. The mitochondrial location of proton-linked MCT1 and LDH-h is highly controversial. Neither has a typical mitochondrial-targeting motif. However, the presence of such a motif would dictate a mitochondrial location and thereby, exclude dynamic sorting of LDH-h and proton-linked MCT1. For example, GAPDH does not possess nuclear- or mitochondrial-localization motifs, yet it translocates to these compartments under certain cellular conditions [11, 63]. In fact, topogenic signals and energy requirements dynamically regulate the localization of the proteins in yeast [64]. Biological processes are guided by economy; the saving of time and energy. For example, the exchange of NADH-H^+^ for NAD^+^ acts as a direct feedback mechanism for the ETC [35]. A direct exchange of mitochondrial LDH-h-generated NADH-H^+^ with the end product of the ETC, NAD^+^, is more efficient and quicker than recovery of cytosolic generated NADH-H^+^ via glycerol-3-phosphate (G3P) shuttle. In the G3P shuttle, pyr^−^ and G3P have to diffuse into the mitochondria and dihydroxyacetone-phosphate has to diffuse back into the cytosol to recover cytosolic NAD^+^. In addition, the overall efficiency is reduced by the fact that mitochondrial G3P dehydrogenase uses the electron carrier flavin adenine dinucleotide (FAD), which generates fewer ATP molecules i.e., less energy. In contrast, time and energy are saved by the LDH-h-driven exchange of NADH-H^+^ for NAD^+^, because l-lac^−^ is substrate of the PDH complex and a carrier of the redox equivalent of one NADH-H^+^ We are aware that pyr^−^ was preferentially sorted into the anabolic pyr^−^-TCA cycle, but here we discuss the G3P shuttle in context of catabolism. It would be of great interest to understand the roles of pyr^−^ and G3P as regulators of lipid synthesis and the formation of FAD-H_2_, perhaps coupled to ETC.

The rationale for connecting enzymes and forming functional enzymes complexes comes from the tentative 4^th^ law of thermodynamics [65, 66] and the proton transport chain hypothesis [35]. The tentative 4^th^ law of thermodynamics was first described in physics and later transferred to economy and biology. It states ‘a flow of energy is sufficient to form ordered structure’ [65]. The proton transport chain hypothesis transfers the 4^th^ law of thermodynamics to biology by using the active H^+^ as the energy entity that orders organisms. In biology, glucose and ATP are stores of energy, like a battery, but the flow of energy is provided by ions, such as H^+^ or Na^+^.

In 1956, Crane described the mechanism of the Na^+^/glucose symporter [67]. This symporter re-absorbs Na^+^ and glucose from gut and pre-urine, by using the flow of Na^+^ (energy) to transport glucose against a concentration gradient. Thus, the Na^+^/glucose symporter produces entropy, a glucose gradient and the flow of energy drives the direction of the transport. It is clear that if the intracellular concentration of Na^+^ were higher than the extracellular, then the same symporter would export glucose [68]. However, in biology, in the setting of an organism, the direction of the Na^+^/glucose symporter is set to act unidirectionally. A similar scenario exists for the mitochondrial ATP synthase complex. For example, if the pH gradient driving the synthesis of ATP were flipped, then theoretically the mitochondrial ATP synthase complex would hydrolyze ATP and pump out protons. However, we prefer to stay within the realms of biology and understand that a flow of energy, in this case, protons, is sufficient to drive the ATP synthase complex and the PDH complex. Thus, investigating isolated enzymes extrudes them from the biological context, disconnecting them from the flow of energy. Isolated enzymes in a test tube reversibly catalyze a concentration equilibrium in a closed system. In contrast, biology describes an open system where energy and material are free to move, i.e., another type of system with different rules.

It is quite interesting that ammonia (NH_3_) or alkalization of the cell, affects the l-lac^−^/pyr^−^ ratio in astrocytes, but not in neurons [59]. From our point of view, the rationale that quenching the activity of pH-sensitive (astrocytic) mitochondrial pyr^-^ transporter is responsible for the effect is critical. This suggests that the membrane transfer of pyr^−^ depends on the pH and not on mitochondrial provision of carbonic acid. This interpretation also suggests that the activity of neuronal pyr^-^ transporter is virtually nil. On the other hand, Na^+^/H^+^ exchangers are well known to regulate cellular pH [69] and in line with our thinking, we predict that these too, transport unidirectionally. The acidifying Na^+^/H^+^ exchanger 1•CAII complex has already been shown to exist, with CAII enhancing exchanger activity [70]. It will be interesting to determine if biology is really simple and that the alkalinizing Na^+^/H^+^ exchanger 3 (*Slc9a3*) [71] forms a complex with PGK.

It might seem strange to develop a hypothesis of schizophrenia, without mentioning dopamine. However, floating down this path we would have to mention the constitutive activity of G protein-coupled receptors. For example, the constitutive activity of dopamine-1-like receptors (D_1_R and D_5_R) increases basal cAMP levels. In contrast, the dopamine-induced effect decreases cAMP levels. Constitutive D_5_R activity increased Na^+^/H^+^ exchanger activity, increased cytosolic pH and accelerated glucose degradation due to increased transcription and translation of the Na^+^/K^+^-ATPase and Na^+^/H^+^ exchanger 2 [72]. So what effect does this balance between constitutive and agonist-induced activity have on cell–cell signaling via neurotransmitters? How is cell homeostasis regulated by constitutive G protein-coupled receptor activity through regulation of glucose metabolism?

Thankfully for scientists, the cell is not just a set of randomly distributed pieces, but a complex puzzle to be solved.

## References

[1] Stöber G, Ben-Shachar D, Cardon M, Falkai P, Fonteh AN, Gawlik M (2009). Schizophrenia: from the brain to peripheral markers. A consensus paper of the WFSBP task force on biological markers. World J Biol Psychiatry.

[2] Maynard TM, Sikich L, Lieberman JA, LaMantia AS (2001). Neural development, cell-cell signaling, and the “two-hit” hypothesis of schizophrenia. Schizophrenia Bull.

[3] Weinberger DR (1987). Implications of normal brain development for the pathogenesis of schizophrenia. Arch Gen Psychiatry.

[4] Gibbs ME (2015). Role of glycogenolysis in memory and learning: regulation by noradrenaline, serotonin and ATP. Front Integr Neurosci.

[5] Rowland LM, Pradhan S, Korenic S, Wijtenburg SA, Hong LE, Edden RA (2016). Elevated brain lactate in schizophrenia: a 7T magnetic resonance spectroscopy study. Transl Psychiatry.

[6] Ho M-W, Ulanowicz R (2005). Sustainable systems as organisms?. Biosystems.

[7] Bornmann L, Mutz R (2015). Growth rates of modern science: a bibliometric analysis based on the number of publications and cited references. J Assoc Inf Sci Technol.

[8] Rapoport SM. Medizinische Biochemie: Lehrbuch für Studierende und Ärzte. Verlag Volk und Gesundheit: Berlin, 1962.

[9] Tompsett SL (1963). Medical biochemistry. Nature.

[10] Azam S, Jouvet N, Jilani A, Vongsamphanh R, Yang X, Yang S (2008). Human glyceraldehyde-3-phosphate dehydrogenase plays a direct role in reactivating oxidized forms of the DNA repair enzyme APE1. J Biol Chem.

[11] Tarze A, Deniaud A, Le Bras M, Maillier E, Molle D, Larochette N (2007). GAPDH, a novel regulator of the pro-apoptotic mitochondrial membrane permeabilization. Oncogene.

[12] Zala D, Hinckelmann MV, Yu H, Lyra da Cunha MM, Liot G, Cordelieres FP (2013). Vesicular glycolysis provides on-board energy for fast axonal transport. Cell.

[13] Sirover MA (2011). On the functional diversity of glyceraldehyde-3-phosphate dehydrogenase: biochemical mechanisms and regulatory control. Biochimica Et Biophysica Acta.

[14] Rapoport SM. Medizinische Biochemie: Lehrbuch für Studierende und Ärzte. 9th ed. VEB Verlag Volk und Gesundheit: Berlin, 1987.

[15] Svedružić ŽM, Odorčić I, Chang CH, Svedružić D. Substrate channeling via a transient protein-protein complex: the case of D-glyceraldehyde-3-phosphate dehydrogenase and L-lactate dehydrogenase. https://www.biorxiv.org/content/10.1101/2020.01.22.916023v1. 2020. 10.1038/s41598-020-67079-2 PMC7320145 32591631

[16] Svedruzić ZM, Spivey HO (2006). Interaction between mammalian glyceraldehyde-3-phosphate dehydrogenase and L-lactate dehydrogenase from heart and muscle. Proteins.

[17] Rogatzki MJ, Ferguson BS, Goodwin ML, Gladden LB. Lactate is always the end product of glycolysis. Front Neurosci. 2015;9. 10.3389/fnins.2015.00022 PMC4343186 25774123

[18] Hebb DO. The organization of behavior: a neuropsychological theory. Wiley: New York, 1949. p. 368.

[19] Brooks GA. Lactate as a fulcrum of metabolism. Redox Biol. 2020;35:101454. 10.1016/j.redox.2020.101454 PMC7284908 32113910

[20] Kim S-Y, Cohen BM, Chen X, Lukas SE, Shinn AK, Yuksel AC (2017). Redox dysregulation in schizophrenia revealed by in vivo NAD+/NADH Measurement. Schizophrenia Bull.

[21] Huh MM, Friedhoff AJ (1979). Multiple molecular forms of catechol-O-methyltransferase. Evidence for two distinct forms, and their purification and physical characterization. J Biol Chem.

[22] Pullen TJ, Sylow L, Sun G, Halestrap AP, Richter EA, Rutter GA (2012). Overexpression of monocarboxylate transporter-1 (SLC16A1) in mouse pancreatic β-cells leads to relative hyperinsulinism during exercise. Diabetes.

[23] Gjedden A, Hansen A, Silver I. The glucose concentration of brain interstitial fluid is low. Proc Int Union Physiol Sci. 1980;14:1553.

[24] Iliff JJ, Wang M, Zeppenfeld DM, Venkataraman A, Plog BA, Liao Y (2013). Cerebral arterial pulsation drives paravascular CSF-interstitial fluid exchange in the murine brain. J Neurosci: Off J Soc Neurosci.

[25] Korogod N, Petersen CCH, Knott GW. Ultrastructural analysis of adult mouse neocortex comparing aldehyde perfusion with cryo fixation. eLife. 2015;4:e05793. 10.7554/eLife.05793 PMC4530226 26259873

[26] Kreft M, Lukšič M, Zorec TM, Prebil M, Zorec R (2013). Diffusion of D-glucose measured in the cytosol of a single astrocyte. Cell Mol life Sci.

[27] Mueckler M, Caruso C, Baldwin SA, Panico M, Blench I, Morris HR (1985). Sequence and structure of a human glucose transporter. Science.

[28] Corvin AP (2010). Neuronal cell adhesion genes: key players in risk for schizophrenia, bipolar disorder and other neurodevelopmental brain disorders?. Cell Adhes Migr.

[29] Keshavan MS, Anderson S, Pettegrew JW (1994). Is schizophrenia due to excessive synaptic pruning in the prefrontal cortex? The Feinberg hypothesis revisited. J Psychiatr Res.

[30] Tadi M, Allaman I, Lengacher S, Grenningloh G, Magistretti PJ (2015). Learning-induced gene expression in the hippocampus reveals a role of neuron-astrocyte metabolic coupling in long term memory. PloS One.

[31] Churchward MA, Tchir DR, Todd KG (2018). Microglial function during glucose deprivation: inflammatory and neuropsychiatric implications. Mol Neurobiol.

[32] Hsieh C-F, Liu C-K, Lee C-T, Yu L-E, Wang J-Y (2019). Acute glucose fluctuation impacts microglial activity, leading to inflammatory activation or self-degradation. Sci Rep.

[33] Steiner J, Bernstein H-G, Schiltz K, Müller UJ, Westphal S, Drexhage HA (2014). Immune system and glucose metabolism interaction in schizophrenia: A chicken–egg dilemma. Prog Neuro-Psychopharmacol Biol Psychiatry.

[34] Holmes E, Tsang TM, Huang JT-J, Leweke FM, Koethe D, Gerth CW et al. Metabolic profiling of CSF: evidence that early intervention may impact on disease progression and outcome in schizophrenia. PLoS Med. 2006;3:e327. 10.1371/journal.pmed.0030327 PMC1551919 16933966

[35] Roosterman D, Meyerhof W, Cottrell GS. Proton transport chains in glucose metabolism: mind the proton. Front Neurosci. 2018;12. 10.3389/fnins.2018.00404 PMC6014028 29962930

[36] Becker HM, Klier M, Schüler C, McKenna R, Deitmer JW (2011). Intramolecular proton shuttle supports not only catalytic but also noncatalytic function of carbonic anhydrase II. Proc Natl Acad Sci USA.

[37] Meyerhof O (1927). Recent investigations on the aerobic and an-aerobic metabolism of carbohydrates. J Gen Physiol.

[38] Zippin JH, Levin LR, Buck J (2001). CO(2)/HCO(3)(-)-responsive soluble adenylyl cyclase as a putative metabolic sensor. Trends Endocrinol Metab.

[39] Roosterman D, Cottrell GS (2020). Astrocytes and neurons communicate via a monocarboxylic acid shuttle. AIMS Neurosci.

[40] Hashimoto T, Hussien R, Brooks GA (2006). Colocalization of MCT1, CD147, and LDH in mitochondrial inner membrane of L6 muscle cells: evidence of a mitochondrial lactate oxidation complex. Am J Physiol Endocrinol Metab.

[41] Dianzani MU (1951). Distribution of lactic acid oxidase in liver and kidney cells of normal rats and rats with fatty degeneration of the liver. Archivio Di Fisiologia.

[42] Baba N, Sharma HM (1971). Histochemistry of lactic dehydrogenase in heart and pectoralis muscles of rat. J Cell Biol.

[43] Das ML, Koike M, Reed LJ (1961). On the role of thiamine pyrophosphate in oxidative decarboxylation of alpha-keto acids. Proc Natl Acad Sci USA.

[44] Reed LJ, Hackert ML (1990). Structure-function relationships in dihydrolipoamide acyltransferases. J Biol Chem.

[45] Bricker DK, Taylor EB, Schell JC, Orsak T, Boutron A, Chen Y-C (2012). A mitochondrial pyruvate carrier required for pyruvate uptake in yeast, Drosophila, and humans. Science.

[46] Herzig S, Raemy E, Montessuit S, Veuthey J-L, Zamboni N, Westermann B (2012). Identification and functional expression of the mitochondrial pyruvate carrier. Science.

[47] Passarella S, Paventi G, Pizzuto R. The mitochondrial L-lactate dehydrogenase affair. Front Neurosci. 2014;8. 10.3389/fnins.2014.00407 PMC4260494 25538557

[48] Pellerin L, Magistretti PJ (1994). Glutamate uptake into astrocytes stimulates aerobic glycolysis: a mechanism coupling neuronal activity to glucose utilization. Proc Natl Acad Sci USA.

[49] Pellerin L (2010). Food for thought: the importance of glucose and other energy substrates for sustaining brain function under varying levels of activity. Diabetes Metab.

[50] Cupello A, Bandini F, Albano C, Favale E, Marchese R, Scarrone S (2008). Catatonic features in major depression relieved by electroconvulsive treatment: parallel evaluation of the status of platelet serotonin transporter. Int J Neurosci.

[51] Underwood EA (1959). John wesley among the physicians: a study of eighteenth-century medicine. Med Hist.

[52] Maurer-Spurej E, Pittendreigh C, Misri S (2007). Platelet serotonin levels support depression scores for women with postpartum depression. J psychiatry Neurosci.

[53] Chen Y, Peng L, Zhang X, Stolzenburg JU, Hertz L (1995). Further evidence that fluoxetine interacts with a 5-HT2C receptor in glial cells. Brain Res Bull.

[54] Hirst WD, Price GW, Rattray M, Wilkin GP (1998). Serotonin transporters in adult rat brain astrocytes revealed by [3H]5-HT uptake into glial plasmalemmal vesicles. Neurochemistry Int.

[55] Silver H, Susser E, Danovich L, Bilker W, Youdim M, Goldin V (2011). SSRI augmentation of antipsychotic alters expression of GABAA receptor and related genes in PMC of schizophrenia patients. Int J Neuropsychopharmacol.

[56] Lynch CJ, Xu Y, Hajnal A, Salzberg AC, Kawasawa YI (2015). RNA sequencing reveals a slow to fast muscle fiber type transition after olanzapine infusion in rats. PLoS ONE.

[57] Fatemi SH (2006). Olanzapine increases glucogenesis by multiple pathways in brain and muscle. Mol Psychiatry.

[58] Chen J, Huang X-F, Shao R, Chen C, Deng C. Molecular mechanisms of antipsychotic drug-induced diabetes. Front Neurosci. 2017;11. 10.3389/fnins.2017.00643 PMC5702456 29209160

[59] Kala G, Hertz L (2005). Ammonia effects on pyruvate/lactate production in astrocytes-interaction with glutamate. Neurochemistry Int.

[60] Lipkovich I, Jacobson JG, Hardy TA, Hoffmann VP (2008). Early evaluation of patient risk for substantial weight gain during olanzapine treatment for schizophrenia, schizophreniform, or schizoaffective disorder. BMC Psychiatry.

[61] Klingerman CM, Stipanovic ME, Bader M, Lynch CJ (2014). Second-generation antipsychotics cause a rapid switch to fat oxidation that is required for survival in C57BL/6J mice. Schizophrenia Bull.

[62] Parker JC, Hoffman JF (1967). The role of membrane phosphoglycerate kinase in the control of glycolytic rate by active cation transport in human red blood cells. J Gen Physiol.

[63] Sawa A, Khan AA, Hester LD, Snyder SH (1997). Glyceraldehyde-3-phosphate dehydrogenase: nuclear translocation participates in neuronal and nonneuronal cell death. Proc Natl Acad Sci USA.

[64] Rojo EE, Guiard B, Neupert W, Stuart RA (1998). Sorting of D-lactate dehydrogenase to the inner membrane of mitochondria. Analysis of topogenic signal and energetic requirements. J Biol Chem.

[65] Jørgensen SE (1999). Tentative fourth law of thermodynamics, applied to description of ecosystem development. Ann N.Y Acad Sci.

[66] Prigogine I (1978). Time, structure, and fluctuations. Science.

[67] Crane RK, Krane SM (1956). On the mechanism of the intestinal absorption of sugars. Biochimica et Biophysica Acta.

[68] Eskandari S, Wright EM, Loo DDF (2005). Kinetics of the reverse mode of the Na+/glucose cotransporter. J Membr Biol.

[69] Murer H, Hopfer U, Kinne R (1976). Sodium/proton antiport in brush-border-membrane vesicles isolated from rat small intestine and kidney. Biochemical J.

[70] Li X, Alvarez B, Casey JR, Reithmeier RAF, Fliegel L (2002). Carbonic anhydrase II binds to and enhances activity of the Na+/H+ exchanger. J Biol Chem.

[71] Turner JR, Black ED (2001). NHE3-dependent cytoplasmic alkalinization is triggered by Na(+)-glucose cotransport in intestinal epithelia. Am J Physiol Cell Physiol.

[72] Roosterman D (2014). Agonist-dependent and -independent dopamine-1-like receptor signalling differentially regulates downstream effectors. FEBS J.

[73] Lowry OH, Passonneau JV (1964). The relationships between substrates and enzymes of glycolysis in brain. J Biol Chem.

